# Orangutans (*Pongo pygmaeus*) Remember Old Acquaintances

**DOI:** 10.1371/journal.pone.0082073

**Published:** 2013-12-04

**Authors:** Yuki Hanazuka, Naoki Shimahara, Yukie Tokuda, Akira Midorikawa

**Affiliations:** 1 Department of Psychology, Graduate School of Letters, Chuo University, 742-1 Higashi-nakano, Hachioji, Tokyo, Japan; 2 Tama Zoological Park, 7-1-1 Hodokubo, Hino, Tokyo, Japan; 3 Department of Psychology, Faculty of Letters, Chuo University, 742-1 Higashi-nakano, Hachioji, Tokyo, Japan; CNR, Italy

## Abstract

Many social animals can discriminate between familiar and unfamiliar faces. Orangutans, however, lead a semi-solitary life and spend much of the day alone. As such, they may be less adept at recognizing conspecifics and are a good model for determining how social structure influences the evolution of social cognition such as facial recognition. The present study is the first report of whether orangutans can distinguish among individual faces. We adopted a preferential looking method and found that orangutans used facial discrimination to identify known conspecifics. This suggests that frequent and intense social interaction is not necessary for facial discrimination, although our findings were limited by the small number of stimuli and the unequal numbers of male and female orangutans depicted in the stimuli.

## Introduction

The ability to distinguish between familiar and unfamiliar individuals is important for social animals. Many social animals, such as humans [1,2], chimpanzees [3,4], rhesus monkeys [5,6], capuchin monkeys [7], dogs [8], sheep [9], cattle [10], and even invertebrates [11] can distinguish between familiar and unfamiliar faces. Previous studies suggest that frequent and intense social interaction may be required for facial recognition. Orangutans, however, are the only species of diurnal primates that have no obvious social groups. They spend the majority of their time alone, with only 5% of their time in social interactions [12]. Recent research reported that Bornean orangutan females spent more time associating with known maternal relatives than with other females [13]. This suggests that orangutans can distinguish specific individuals. Additionally, it is assumed that orangutans avoid mating with close relatives. However, little is known about how orangutans recognize conspecifics. Are orangutans as good at distinguishing between familiar and unfamiliar faces as are other social species? Additionally, are they able to remember and discriminate historically familiar faces that they have not seen for a long period of time? 

Operant conditioning is often used to determine cognitive ability in animals because it allows detailed analysis of the ability to distinguish between different stimuli. However, operant conditioning is unsuitable for determining natural cognition used in everyday life because the training may influence and possibly improve innate abilities [14]. The preferential looking method allows examination of natural cognitive function without interference from training. This method was developed to examine perceptual function in human infants [15], but it has also been applied to chimpanzees [16], gibbons [17], rhesus monkeys [5], dogs [8], and rooks [18]. To examine the facial recognition ability of orangutans, we simultaneously presented two images of faces to participants. If the individuals were able to distinguish between familiar and unfamiliar faces following this protocol, we would be able to conclude that orangutans have a natural ability for facial recognition.

## Results and Discussion

**Figure 1:**
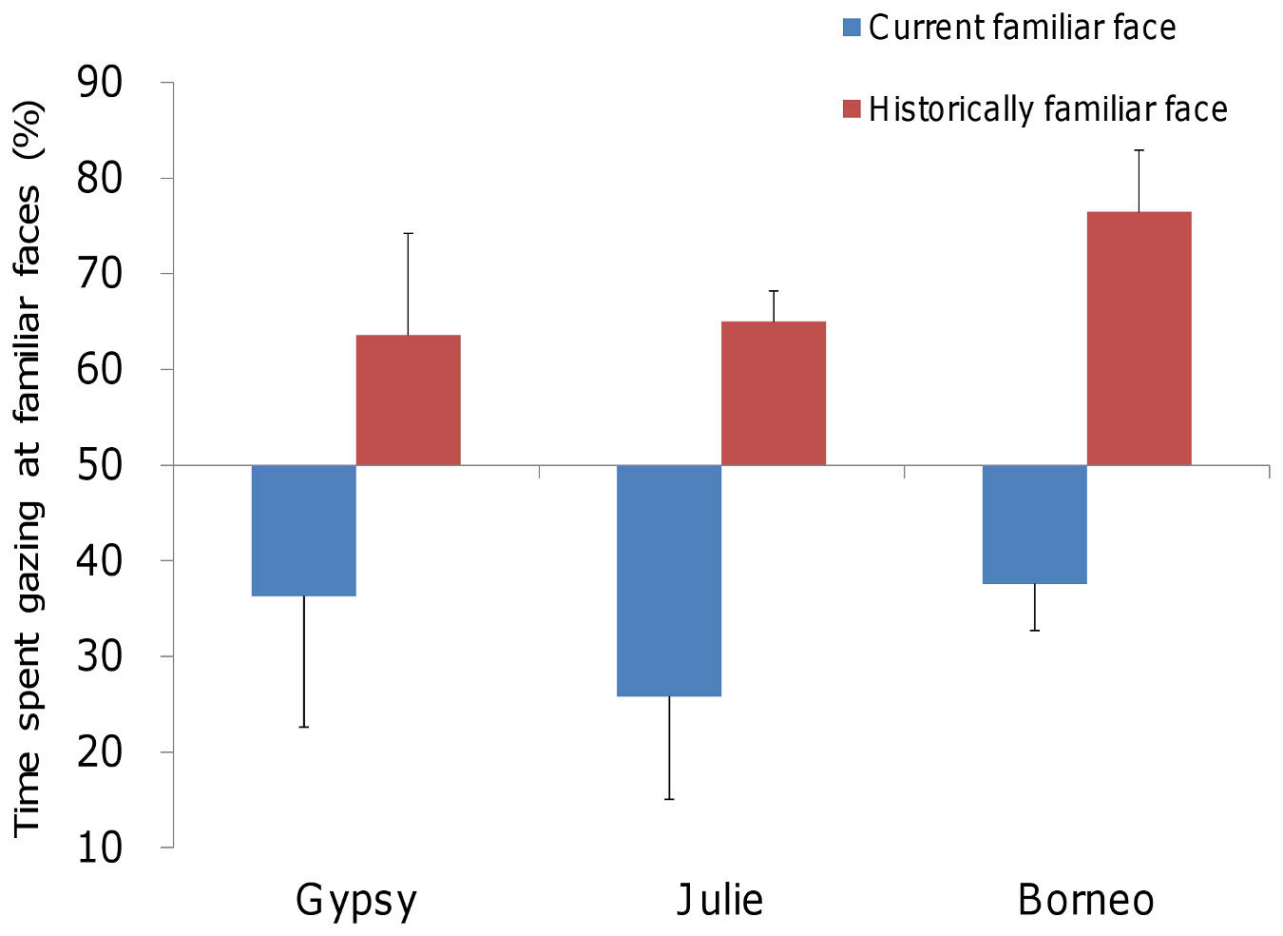
Mean (± SEM) preference scores for familiar faces. The preference scores were calculated as percentages. Blue bars indicate the percentage of time spent gazing at currently familiar faces, and red bars indicate the percentage of time spent gazing at historically familiar faces. Gypsy, Julie, and Borneo are the orangutans used in this study.

Under the currently familiar face condition (CF), a significant preference for unfamiliar faces was observed (two-tailed *t* test vs. chance; Gypsy: *t* (7) = 2.69, *p* < 0.05; Julie: *t* (7) = 3.87, *p* < 0.01; Borneo: *t* (5) = 2.69, *p* < 0.05; Figure 1 blue bars). In contrast, under the historically familiar face condition (HF), the reverse pattern was found, and a significant preference for historically familiar faces was observed (two-tailed *t* test vs. chance; Gypsy: *t* (5) = 3.21, *p* < 0.05; Julie: *t* (5) = 6.78, *p* < 0.01; Borneo: *t* (5) = 8.41, *p* < 0.01; Figure 1 red bars). The response patterns of orangutans were stable during the experimental period. Significant differences between the first and second trials were not seen in any subject (CF condition: Gypsy, *t* (6) = 0.53, *n.s.*; Julie: *t* (6) = 0.81, *n.s.*; Borneo: *t* (4) = 0.46, *n.s.*, HF condition: Gypsy, *t* (4) = 0.13, *n.s.*; Julie: *t* (4) = 1.13, *n.s.*; Borneo: *t* (4) = 0.68, *n.s.*). 

In the wild, female Bornean orangutans spend more time associating with known maternal relatives than with others [12], but the clues used by orangutans to discriminate among individuals have not been understood. Our results demonstrate that orangutans use facial discrimination to identify known conspecifics. This ability may help orangutans decide whether to approach or avoid the individuals they encounter. Like other social species, orangutans can discriminate among faces even though they have less social interaction than do other species. 

It has been assumed that orangutans, like many animals, avoid mating with close relatives [19]. The average life span of orangutans is approximately 50 years [20]. Over such a long lifespan, individuals should have many opportunities to encounter their conspecifics. Our results suggest that the ability to discriminate between historically familiar and unfamiliar faces may be useful for enabling orangutans to discriminate between relatives and strangers. 

Interestingly, when unfamiliar- familiar faces were paired, orangutans paid more attention to unfamiliar faces, whereas when unfamiliar-historically familiar faces were paired, they paid more attention to historically familiar faces. This may indicate different cognitive processes for recognizing currently and historically familiar faces. Preferential viewing studies of monkeys [5] and human infants [21] have consistently found preferences for unfamiliar (novel) faces. In contrast, other studies have shown preferences for familiar faces that the individual had not seen in a long time [6,22]. Therefore, orangutans show the same facial preferences as do other primates, including humans. 

However, the present study had several limitations. First, the small number of stimuli used limited the generalizability of our findings. Moreover, we used only one picture of each stimulus individual, which could have unintended effects because orangutans may attend to physical attributes in addition to luminance and color. Multiple pictures of each stimulus individual should have been prepared to verify that the effect was consistent across different pictures of the same stimulus individuals. Second, the stimuli depicted an unequal number of male and female orangutans. Thus, one possible interpretation of our data is that rather than showing a preference for unfamiliar faces (as opposed to currently familiar faces), the orangutans were avoiding looking at known dominant males. Similarly, the orangutans may not have preferred a historically- familiar over an unfamiliar male. Thus, gender, rather than familiarity, may have influenced the orangutans’ preferences.

In terms of comparative cognition, facial recognition in orangutans was similar to that observed in humans. Humans can recall faces of high school classmates after a quarter of a century [23]. The ability to recognize historically familiar faces may be shared by humans and orangutans. Further comparative examination of face recognition is necessary for understanding the mechanisms underpinning this ability in both humans and other primates. 

## Materials and Methods

### Ethics

Subjects participated voluntarily in the study and were never deprived of food or water. The research was non-invasive and conducted in resting rooms. To reduce stress to the subjects, we conducted the experiments within 20 minutes. The Tama Zoological Park Ethics gave full ethical approval to this behavioral, non-invasive study, which complied with the code of ethics of the Japanese Association of Zoos and Aquariums (JAZA). Animal husbandry and research complied with the ''WAZA Ethical Guidelines for the Conduct of Research on Animals by Zoos and Aquariums”.

### Participants

The participants were Bornean orangutans housed at the Tama Zoological Park in Tokyo, Japan. The sample consisted of two females (Gypsy, approximately 55 years old, and Julie, 45 years old) and one male (Borneo, 25 years old). Gypsy was born in the wild and has lived at Tama Zoological Park for over 50 years. Julie was born and reared in Tama Zoological Park, and Borneo was born in the Singapore Zoo and moved to Tama Zoological Park at 1998. Only Gypsy had previously participated in experiments testing cognitive abilities [24]. Julie and Borneo were naïve with regard to computational tasks. Gypsy and Julie were housed socially and had direct and visual contact with other individuals. Borneo also had the opportunity to see other individuals but was housed separately. All orangutans were fed daily with fruits and vegetables, and received water *ad libitum*. They sometimes received cloths and magazines as enrichment tools. 

### Apparatus

The orangutans were tested in their resting room (1.8 m wide × 2.6 m deep × 2 m high), which is surrounded by a mesh fence (each hole in the mesh measures 5 × 5 cm). The stimuli were presented using two 17-inch liquid crystal monitors (LCD-A173KB, IODATA) placed diagonally with respect to each other (150°angle) and controlled by a laptop computer (SL500 2746-7DJ, ThinkPad). The distance between the participant and the monitors was approximately 60 cm. A video camera (DCR-HC62, Sony) on a tripod was placed between the monitors and the laptop to record the gaze of each orangutan.

### Stimuli

**Figure 2:**
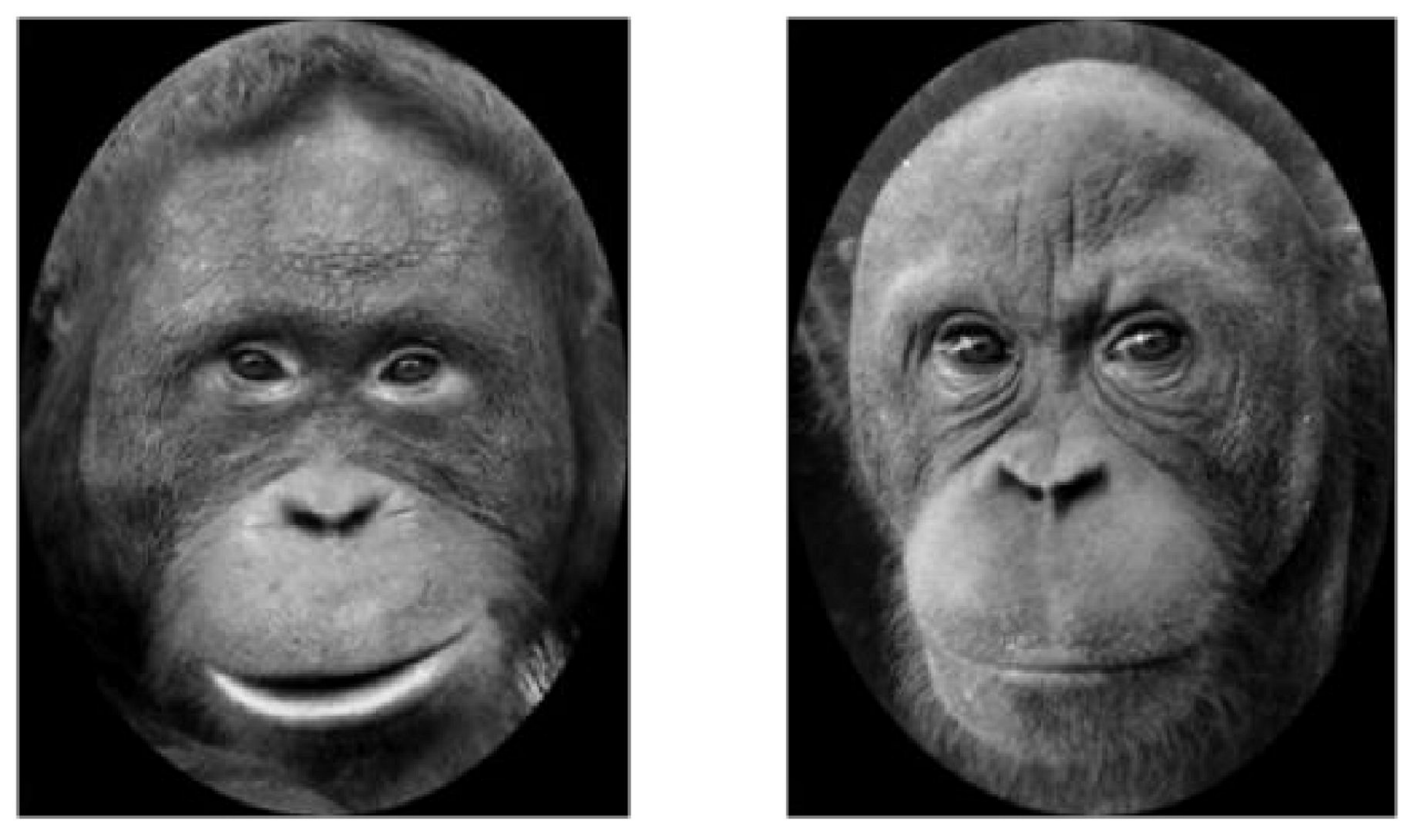
Examples of familiar (left) and unfamiliar (right) faces used in this experiment.

We prepared three sets of stimuli that differed in familiarity. The set of currently familiar faces (CF) consisted of facial images of four conspecifics the orangutans saw two or three times a week. Only Borneo was presented with three pairs under the currently familiar face condition because the stimulus set contained photographs of Borneo himself. The set of historically familiar faces (HF) consisted of facial images of three conspecifics the orangutans had seen 10 years ago. Gypsy and Julie had lived historically with individuals (Sally, Kewpie, and Yully) and had had the opportunity to see them daily for 10 years. On the other hand, Borneo had had the opportunity to see Kewpie every day and to see Sally and Yully once a month through a grid. The third set was of facial images of seven unfamiliar individuals the orangutans in the present study had not seen before. These unfamiliar faces were equivalent to the familiar faces in terms of gender and age. Only one picture of each individual was used in this experiment. All images were 17 cm × 21.7 cm digitized gray-scale images (640 × 824 pixels) created from color photographs and videos via the GNU Image Manipulation Program (Free Software Foundation, Inc.). The luminance of the stimuli was adjusted to 81.4-87.0. A uniform black background was imposed around each facial image to maintain homogeneity of the stimuli (Figure 2). Information about the stimulus individuals is set out in Table 1 and 2. 

**Table 1:** Information about current familiar individuals.

			Blood relation to the experimental subjects		
Photograohic subjects	Gender	Age	Gypsy	Julie	Borneo
Kyu	M	41	non-kin	non-kin	non-kin
Chappy	F	37	Daughter	sister	non-kin
Borneo	M	25	non-kin	non-kin	self **^*a*^**
Poppy	M	10	Grandson	sister's son	son

^a^ The trials of Borneo were excluded from the analysis for Borneo.

**Table 2:** Information about historically familiar individuals.

			Blood relation to the experimental subjects		
Photograohic subjects	Gender	Age	Gypsy	Julie	Borneo
Sally	F	32	Daughter	sister	non-kin
Kewpie	F	17	Granddaughter	sister's daughter	non-kin
Yully	F	8	Granddaughter	sister's daughter	non-kin

### Procedure

The experiment began when an orangutan voluntary sat in front of the apparatus. Stimuli were shown in Hot Soup Processor 3.0 (ONION software). At the beginning of each trial, a photograph of fruit (apple, orange, or strawberry) with a brief sound was presented at the center of the monitor to attract the orangutan’s attention. The experimenter initiated each trial as soon as the orangutan began paying attention to the photograph. In each trial, familiar and unfamiliar faces were presented side by side on the two monitors. The stimuli were continuously presented until the orangutan looked at either image for more than 5 seconds, at which point a new set of faces was presented. Seven trials (CF condition and HF condition) were conducted within the same session, and two sessions were conducted once a month to prevent the orangutans from habituating to the stimuli. Thus, the orangutans participated in eight trials under the CF condition and six trials under the HF condition (only Borneo participated in six trials under the CF condition). The order of stimuli was randomized per session and the position of the stimuli (left or right side) was counterbalanced between sessions. 

### Data Analysis

Codes were assigned with an accuracy of 33 ms (per video frame) using Edius Pro4 (Grass Valley K.K). We measured the total time looking at each side. One of the authors (YH), coded the orangutans’ gaze directions into three categories: looking at the left monitor, looking at the right monitor, or not looking at either monitor. To ensure the reliability of judging, a second scorer analyzed 20% of all trials. The first and second scorers had no knowledge of the stimuli presented on the display when they assessed the orangutan’s looking behavior. According to Cohen’s *kappa*, the level of inter-judge reliability in this study was relatively high (κ = 0.84). Because the time of each test varied, we calculated the proportion of time spent viewing the left and right images. For each orangutan, we performed a t-test to compare looking preferences against chance level (50%), treating each trial as a data point.
